# Characterization of antiviral resistance mutations among the Eastern Indian Hepatitis B virus infected population

**DOI:** 10.1186/1743-422X-10-56

**Published:** 2013-02-14

**Authors:** Rajesh Panigrahi, Avik Biswas, Binay Krishna De, Sekhar Chakrabarti, Runu Chakravarty

**Affiliations:** 1 ICMR Virus Unit, Kolkata, ID & BG Hospital Campus, Kolkata, India; 2 Department of Medicine, Calcutta Medical College, Kolkata, West Bengal, India; 3 National Institute of Cholera and Enteric Diseases, Kolkata, India

**Keywords:** HBV infection, Drug resistance, Drug-naïve patients, Eastern India

## Abstract

**Background:**

Antiviral therapy using nucleos(t)ide analogues (NAs) is an effective control measure of chronic hepatitis B virus (HBV) infection; however they need long term treatment**.** Presence of drug-resistance mutations may get in the way of the efficacy of antiviral therapy. Our study was aimed at defining the prevalence of HBV drug-resistance in HBVrt region in a population of 147 HBsAg positive patients.

**Findings:**

HBV/D has shown multiple types of HBVrt mutations both among treatment naïve (65.0%, 13 of 20 HBV/D) and treated patients (56.2%, 9 of 16 HBV/D). In additional, several mutations, with a suggested role in drug resistance, were detected among the treatment naïve as well as the treated patients. The mutations reported to be involved in reduction of drug effectiveness, was common among non-responders to therapy as well as among the naïve patients. Notably, classical antiviral resistance mutations (rtL80I/V-rtI169T-rtV173L-rtL180M-rtA181T/V/S-rtT184A/S/G/C-rtA194T-rtS202C /G/I -rtM204V/I-rtN236T-rtM250V) were not detected.

**Conclusion:**

The prevalence of putative NAr mutations among non responders to therapy suggests that they might have role in reduced efficacy of currently available antivirals and requires further investigations.

## Introduction

Chronic hepatitis B virus (HBV) infection remains a serious public health problem in India. Antiviral therapy using nucleos(t)ide analogues (NAs) is an effective control measure; however they need long term treatment. The limited numbers of NAs available and development of drug resistance conferred by viral mutations in the HBV reverse transcriptase (rt) domain during long term treatment remains the major concern leading to treatment failure [1]. Recent reports showed presence of antiviral resistance even in HBV isolates from therapy naive patients [2-4].

Mutations reported in response to available NA therapy for HBV is defined as primary mutations, which reduces HBV replication fitness [5]. Secondary/compensatory mutations develop subsequently that restore the replication capability of HBV [6]. Genotype-dependent polymorphic amino acid (aa) positions were recognized in the HBVrt that may influence the development of drug resistance. Recently, HBVrt domain mutations which have been reported in the literature as supposed drug resistant mutations but are not verified experimentally were classified as putative NA resistant (NAr) mutations (such as S53N, T54N, L82M, V84M, S85A, I91L, Y126C, T128I, T128N, N139D, W153Q, F166L) [7]. Thus, the HBVrt domain mutations can be classified into four categories (primary drug resistance mutation, secondary/compensatory mutation, putative NAr mutation and pretreatment mutations). Pretreatment mutations (such as T38A, Y124H, D134E, N139K/H, I224V, and R242A) were defined as amino acid substitutions that have been reported among NA-naive patients but their relationships with antiviral resistance development have not been clarified yet.

Eight HBV genotypes (A–H) and several subgenotypes within certain HBV genotypes have been identified [8], with distinct geographical distributions. Eastern India is a unique region where three different HBV genotypes (A, C and D) co circulate among the same ethnic group. The rate of HBV drug resistant strains in Eastern Indian patients is still poorly defined. In this cross sectional study, our focus was to analyze the prevalence of HBV/rt region mutations in patients from Eastern India.

## Materials and methods

Blood samples obtained from 147 HBV surface antigen (HBsAg) positive patients referred to our unit were included in this retrospective study. The inclusion criteria were hepatitis B surface antigen (HBsAg) positive; exclusion criteria were hepatitis C virus or human immunodeficiency virus co-infection. Among them HBVrt region could be successfully amplified and sequenced from 85 samples (55 antiviral therapy naïve patients and 30 non-responder to NA therapy). The low amplification of the samples may be due to the low copy number of HBV DNA in the plasma. The treatment drug selection was lamivudine and combinational therapy (adefovir and lamivudine). The serology of the patients was done as described previously [9]. The selection of non-responder patients were on the basis of the failure to achieve more than 1 log10 decrease from base-line within 6 months of starting therapy [10]. The study was part of a project, approved by the Institutional Ethics Committee NICED (ICMR) and informed written consent was obtained from all the subjects and was funded by CSIR, New Delhi.

Nested polymerase chain reaction was used to amplify HBV RT region using primers (HB1F-5^′^-AAGCTCTGCTAGATCCCAGAGT-3^′^ (sense 18 to 40); HB6R 5^′^-AACAGACCAATTTATGCCTA-3^′^ (antisense 1809 to 1790) in 30 μl reaction volume and 4 μl from the first round product was amplified using primers B2 5^′^- GGCTCACAGTTCACGGAACAGT-3^′^(sense 65 to 86) and HS4R 5^′^- CATACTTTCCAATCAATAGG-3^′^(antisense 992 to 973) and a standard thermal cycling profile, with annealing 55°C for 45 s, 45 cycles (40 cycles for the second round).

The amplicons were subjected to direct sequencing and the data were analyzed as previously described [9]. HBV genotypes were assigned using NCBI Viral Genotyping Tool (http://www.ncbi.nlm.nih.gov/projects/genotyping/formpage.cgi) and phylogenetic analysis with MEGA 4.0 software. Multiple clonal analyses of the samples were done as described previously from our laboratory [11]. For statistical calculations, StatCalc (EpiInfo, v 6.0, CDC, USA program is used. For the purpose of our study, a ‘p’ value less than or equal to 0.05 was considered statistically significant. Gene Bank Accession numbers: JQ316695-JQ316779.

## Result

Among the 85 DNA positive subjects whose HBV RT sequences were determined, 70 (82.4%) were male. Of the treated patients the 12/30 was found to be HBeAg positive as compared to 28/55 HBeAg positive among the naive patients. Prevalent HBV genotypes were similar to that previously reported from eastern India. The main characteristics of the treated and naïve patients were compared in Table 1.

**Table 1:** Main characteristics of the study population

**Characteristics**	**Treatment naïve (N= 55)**	**Antiviral treated (N= 30)**
Sex (male/female)	46/9	24/6
Age (years)#	33.5±12.2	31.2±12.0
ALT level [U/L]#	87.7±76.1	60.8±23.3
Genotype (A/C/D)	22/13/20	4/10/16
Pol Gene Amplified	55	30
HBV-DNA (log_10_ copies/ml)#	5.07±1.02	4.17±1.74

# Mean ± SD (standard deviation).

Whole genome sequences of a particular genotype retrieved from GenBank were used to produce a consensus sequence of the whole polymerase gene and were aligned with the study samples and different mutations and substitutions were analyzed. Three HBV genotypes were found, with a prevalence of HBV/A (26/85; 30.5%), HBV/C (23/85; 27.0%) and HBV/D (36/85; 42.3%). Compared to HBV/A and HBV/C genotypes, HBV/D has shown multiple different types of HBVrt mutation patterns (Putative NAr mutation and Pretreatment mutation) both among naïve and treated patients as 65.0% (13/20) and 56.2% (9/16) respectively (Table 2). The difference in the prevalence of HBVrt mutation patterns among the naïve and treated patients was insignificant (p=0.9; 34.5% and 36.67% respectively).

**Table 2:** Distributation of HBVrt mutations among the three genotypes

	**Antiviral treated (N= 30)**			**Treatment naïve (N= 55)**		
**GENOTYPES→**	**HBV/A (4)**	**HBV/C (10)**	**HBV/D (16)**	**HBV/A (22)**	**HBV/C (13)**	**HBV/D (20)**
**Putative NAr mutation**	Y126H ***(4)***, N139E/Q ***(4)***, F221Y***(4)***	I91L ***(9)***, Y126H ***(10)***, W153R ***(10)***,	I91L ***(15)***, Y126H ***(11)*** Y126R ***(5)***, W153R ***(16)***, S213T ***(1)***, Q215S ***(1),*** Q215P ***(1)***,	Y126H ***(17)***, N139E ***(1)***, N139Q ***(21)***, V191I ***(1)***, F221Y ***(22)***,	I91L***(12)***, Y126H ***(13)***, W153R ***(13)***, V191F ***(1)***, Q215E ***(1)***,	I91L ***(20)***, Y126R ***(13),*** Y126H ***(7)***, T128I ***(1)***, W153R ***(20)***, Q215H ***(1)***, Q215S ***(1)***, F221Y ***(1)***, L229F ***(1)***
**Pretreatment mutation**	N139E/Q **(4),** I224V **(4)**		Y124H **(15)**, I224V **(16)**	Y124D **(1)**, Y124N **(21),** N139E **(1),** N139Q **(21),** I224V **(22)**	Y124H **(1),** D134E**(3),** I224V **(1)**	Y124H (19), Y124N(1), D134E(1), I224V(20)

Values in the parentheses indicates number of isolates.

**Figure 1:**
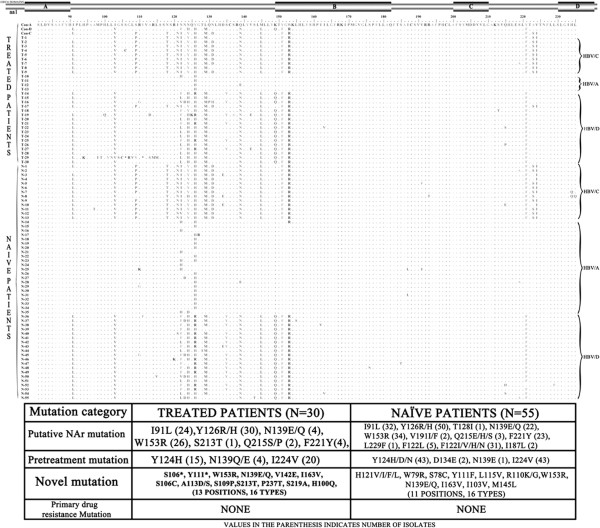
**Genotype associated and independent amino acid mutations in the HBVrt domain among the antiviral drug treated and naïve patients.** The four domains of the HBVrt region are indicated by horizontal bars (above). The amino acid alignment of the domain among the antiviral drug treated and treatment naïve patients are shown and the genotype of each isolate are indicated in the right side.

HBV RT region consists of 6 functional domains (F, A, B, C, D and E) and 5 interdomains (F–A, A–B, B–C, C–D and D–E) [12,13]. Analysis of the domains from naïve patients revealed the presence of 16 different mutations, whereas domain C was conserved (Figure 1). When the interdomains were analyzed a total of 18 mutations were found including a conserved D-E interdomain. In addition, the A–B interdomain displayed the most abundant mutations indicating these positions might be naturally occurring mutation hotspots. The known lamivudine (LMV) resistant mutation rtI187L was found in two therapy naïve patients. Analysis of pol gene mutations with respect to the genotype distributation showed that rtI91L and rtV103I was found among the HBV/A and HBV/D but not in HBV/C. All the isolates having rtI91L were associated with rtV103I. Notably, mutations, recently proposed as NAr mutations i.e. which are potentially associated in reduction of drug effectiveness, were found in the naïve patients.

Among the treated patients no primary antiviral drug resistant mutations, confirmed by in vitro studies as associated with resistance to NA (rtL80I/V-rtI169T-rtV173LrtL180M-rtA181T/V/S-rtT184A/S/G/C-rtA194T-rtS202C/G/I-rtM204V/I-rtN236T-rtM250V) were found. The prevalence of rtI91L was 80% (24/30) among them. Several other pretreatment mutations were also found in the treated patients (Figure 1). Two stop codon mutations were identified at position rtS106* and rtY111* in HBV/D genotype and both was found to be present as unique viral variant.

## Discussion

In this study several genotype-dependent aa polymorphic positions were identified for HBV/A, HBV/C and HBV/D genotypes (Table 2). Our study showed that rtI91L was favored in HBV/C and HBV/D. This mutation have been reported to be more common among cases with extended treatment failure than rtI91, and suggested as potential pretreatment markers to predict the long term response to LMV therapy [7]. In addition, Y126H was more frequent among treatment non responders infected with HBV/D (68.75%, 11/16) than among treatment naïve patients infected with HBV/D (35%, 7/20; Table 2), although not statistically significant (p=0.2). Whereas, in patients infected with HBV/C or HBV/A, Y126H is common among both naïve and treated patients. The potential role of HBV genotypes on modulating resistance development is still disputed [14]. A recent study by Svicher et al. (2009) [14] has demonstrated that genotypes A and D had different preferences for two distinct LMV-resistance mutation clusters, underscoring possible roles of HBV genotypes in driving RT sequence evolution under NA treatment. The present study also shows a high HBVrt polymorphism with potential role in NA resistance, seen in treatment naïve patients with HBV/D. This is clinically significant in India, with a higher burden of chronically infected individuals with predominance of HBV/D and extensive use of LMV [15]. Recent study from Spain [16] showed a lower prevalence of HBVrt mutations among the HBV/D. This is in contrast to a higher prevalence of HBVrt mutations, found in our study. Notably, they have studied mainly well-known primary NA resistant mutations and a few potential drug resistant mutations. In contrast our study, analyzed all 4 types of the possible NAr mutations. Additionally, this might also be due to altered mutational profile because of HBV/D subgenotype variations between the two geographic regions; as discussed earlier [17]. Therefore, in regions where more than one genotype/subgenotype is prevalent, genotype/subgenotype associated polymorphism in the RT domain affecting NA resistance data needs further study.

The probability of selecting antiviral resistance is dependent on the intensity of selection pressure and the diversity of HBV quasispecies [18]. Among the naive patients in our study, treatment was not the selection pressure for developing mutations. Rather, mutations might have been driven by the patient’s immune system conferring an advantage to the wild-type virus. This is supported by clustering of mutations in the A–B interdomain, corresponding to the overlapping ‘a’ determinant of HBsAg, which is under high host immune pressure; thus this region is not likely to be important for RT function and antiviral resistance.

In our study group, putative NAr mutations were found among the therapy naïve patients too. The therapy naive patients might have been infected with strains from other patients who had been treated with NA or these might be host immune pressure driven mutations. The dynamics of emerging NA resistance mutations in untreated patients should be the result of both viral factors and host factors. However, presence of pretreatment variants in NA-naive patients might also be the result of transmission of mutant strains from patients or due to natural variation of HBV. We presume that those positions in the functional domains or adjacent to the classical mutation positions might have significance and needs further consideration (Figure 1).

In India, LMV is widely used and adefovir is an alternative drug of choice. Therefore, the possibility of detecting pre-existing HBV variants resistant to these drugs is more when compared to other drugs introduced rather recently. In the present study, no established primary mutations related to antiviral drugs (rtL80I/V-rtI169T-rtV173L-rtL180M-rtA181T/V/S-rtT184A/S/G/C-rtA194T-rtS202C/G/I-rtM204V/I-rtN236T-rtM250V) were detected neither among the therapy naïve patients nor among the non-responders to NA therapy. Although putative mutations widely found in treatment naive patients would suggest that they may not be wholly responsible for non-response to NA therapy; however, this needs confirmation by *in-vitro* and follow up studies. As per the statement of the patients, they have never missed the drug. However no pill count was done, therefore the question of patient’s compliance cannot be ruled out. Therefore, the role of putative NAr mutations on nonresponse to antivirals requires further *invitro* and follow-up studies.

In conclusion, in our study classical antiviral resistance mutations (rtL80I/V-rtI169T-rtV173L-rtL180M-rtA181T/V/S-rtT184A/S/G/C-rtA194T-rtS202C/G/I-rtM204V/I-rtN236T-rtM250V) were not detected both in naïve patients and NA therapy non responders infected with any of the three HBV genotypes present. the prevalence of putative NAr mutations among the study subjects suggest that they might have role in reduced efficacy of currently available therapy, but their impact on antiviral efficacy requires further *invitro* and follow-up studies. Thus, routine use of resistance testing in patients before initiating antiviral therapy is not necessary at present.

## Competing interests

The authors declare that they have no competing interests.

## Authors’ contributions

RP performed the majority of experiments; AB and RC were involved in experiment design; BKD, SC and RC participated in designing the study, preparation and editing the manuscript. All authors have read and approved the final manuscript.
